# Electrotransport Properties of Perfluorinated Cation-Exchange Membranes of Various Thickness

**DOI:** 10.3390/membranes13110873

**Published:** 2023-11-03

**Authors:** Irina Falina, Natalia Loza, Marina Brovkina, Ekaterina Titskaya, Sergey Timofeev, Natalia Kononenko

**Affiliations:** 1 Physical Chemistry Department, Faculty of Chemistry and High Technologies, Kuban State University, 350040 Krasnodar, Russia; nata_loza@mail.ru (N.L.); andreeva_marina_90@bk.ru (M.B.); katezolotka@mail.ru (E.T.); kononenk@chem.kubsu.ru (N.K.); 2 JSC Plastpolymer, 195197 Saint Petersburg, Russia; svtimof@mail.ru

**Keywords:** proton exchange membrane fuel cell, perfluorinated sulfonic cation-exchange membranes, thickness, conductivity, diffusion permeability, current–voltage curve

## Abstract

The present work discusses the influence of the thickness of MF-4SK perfluorinated sulfonic cation-exchange membranes on their electrotransport properties in hydrochloric acid solutions. It is found that diffusion permeability and conductivity are primarily determined with the specific water content of the membranes and increase with their increase. Analysis of the contribution of reverse diffusion through the membrane to the value of the limiting current shows that it can reach 20% for membranes with a thickness of 60 μm. A study of the characteristics of the fuel cell with perfluorinated membranes of different thicknesses shows that the membrane thickness affects both the ohmic resistance of the membrane-electrode assembly and the diffusion limitations of proton transport in polymer electrolytes.

## 1. Introduction

Polymer ion-exchange membranes are widely used in proton exchange membrane water electrolyzers (PEMWE) and electrochemical energy sources, such as proton exchange membrane fuel cells (PEMFC) and aqueous redox-flow batteries (RFB). The main requirements for polymer materials for these devices are high ion conductivity; stability in redox environments and at elevated temperatures; mechanical strength in dry and swollen state; low permeability to the fuel used (hydrogen, methanol, etc.); and relatively low cost [1,2,3,4,5,6,7,8]. The main difference between the operating conditions of RFB membranes compared to PEMWE and PEMFC is that they operate in an aqueous solution containing the oxidizing and reducing agents, while in PEMWE and PEMFC the membrane operates under conditions of limited humidity and elevated temperature. An additional requirement for PEMFC is the ability to retain water under operating conditions to ensure high proton conductivity, while membranes for RFB with organic redox agents should be able to resist fouling of the membrane with redox components [7].

Compliance with the listed requirements is determined with the chemical structure of the elementary unit of the polymer membrane. The membrane resistance to radical oxidation (Fenton test) increases with the increasing degree of fluorination of the polymer [9]. As a rule, several groups of fluorinated polymers are used for these purposes: aliphatic non-cross-linked or aromatic highly cross-linked polymers/copolymers; for example, polybenzimidazoles, polyether ether ketone, polystyrene, etc. [1,2,3]. To ensure proton conductivity, sulfonic or phosphonic acid groups are introduced into their composition. Operating under oxidative conditions leads to the destruction of polymers. Taking into account the above requirements, today the best properties for use in the mentioned fields are possessed by perfluorinated polymer systems, which include a number of commercial polymers: Nafion (DuPont, Wilmington, DE, USA), Flemion (Asahi Glass Co., Ltd., Tokyo, Japan), Aquivion (Solvay SA, Brussels, Belgium), 3M (3M India Ltd., Karnataka, India), Fumion/Fumapem (FUMATECH BWT GmbH, Bietigheim-Bissingen, Germany), and MF-4SK (JSC “Plastpolymer”, St. Petersburg, Russia). Currently, the undisputed leader among these materials is the perfluorinated Nafion membrane.

A special requirement for membranes for the mentioned applications is their low thickness, which reduces the overall cell resistance. Nowadays, insufficient attention is paid to the patterns of the influence of membrane thickness on its transport characteristics, which is of fundamental importance. An increase in the thickness of the polymer electrolyte leads to an increase in the resistance of the MEA and a decrease in the fuel crossover [10]. An increase in crossover leads to the growth of hydrogen peroxide formation in PEMFC and PEMWE [6], which appears in the destruction of the polymer and side chains during operating the device in severe conditions (especially at potentials close to the open circuit potential). It is indicated by a decrease in ion-exchange capacity [11,12] and the formation of cavities on the surface of membrane [13,14]. These processes cause physical degradation of the membrane due to inelastic deformations, the formation of defects, and reorganization of the polymer structure [15], which causes an increase in its gas permeability and a reduction in the characteristics of the cell.

Thickness could also affect the electrical conductivity of the membrane. The thickness of the polymer electrolyte affects the redistribution of water in its volume [15]. Thus, an increase in membrane conductivity both in a 1 M sulfuric acid solution [16] and at elevated temperature and restricted humidity [17] with increasing sample thickness is noted. When studying the conductivity of membranes under limiting humidity [18], an increase in the conductivity of samples with increasing membrane thickness, which consists of volume and surface conductivity, was also found [2,19].

The present work discusses the influence of thickness of perfluorinated cation-exchange membranes with sulfonic acid groups on conductivity, diffusion permeability, and current–voltage curves in hydrochloric acid solutions and the characteristics of MEA.

## 2. Materials and Methods

### 2.1. Materials

The objects of research are the experimental samples of perfluorinated MF-4CK membranes with sulfonic acid groups (JSC “Plastpolymer”, St. Petersburg, Russia). The series of studied membranes includes samples of various thicknesses obtained via extrusion and casting. The perfluorinated Nafion NRE-212 membrane (Du Pont, Wilmington, DE, USA) is also studied. 

### 2.2. Experimental Techniques

#### 2.2.1. Physical–Chemical Characteristics

The ion-exchange capacity (IEC) was determined according to the next procedure: the membrane sample with a definite weight in H^+^-form was immersed in a mixed solution of KOH and KCl. The decrease in KOH concentration after contact with the membrane was determined via titration and used for IEC calculation. The water content (W_dry_), which is the weight of water that could be absorbed by the membrane, was measured via air-heat drying. Specific water content (n_m_), which is the number of water molecules per sulphonic group, was calculated from IEC and W_dry_ values.

#### 2.2.2. Diffusion Permeability and Conductivity

The diffusion permeability was measured for the membrane in a freestanding state under the diffusion of a hydrochloric acid solution with a defined concentration through the membrane into distilled water [20]. The increase in electrolyte concentration in the chamber, initially filled with distilled water, was monitored via conductometry using platinum electrodes connected to an E 7-21 RLC-meter (MNIPI, Minsk, Republic of Belarus).

The specific conductivity of the membranes was determined based on their active AC resistance, measured with the mercury-contact method using potentiostat-galvanostat P-45X (Electrochemical Instruments, Chernogolovka, Russia) equipped with the FRA-24M impedance measurement unit [21]. The peculiarity of the method is using liquid mercury electrodes, which provides good contact between the membrane and the electrodes while the membrane is in a freestanding state.

#### 2.2.3. Measurements of Current–Voltage Curves

The experimental setup for the measurements of current–voltage curves (CVCs) of studied membranes in “free-standing state” comprised a four-compartment cell with a four-electrode arrangement using a 0.05 M HCl solution [22]. The anion-exchange MA-41 and cation-exchange MF-4SK membranes were used as auxiliary ones. The MA-41 membrane consists of an anion exchange resin with quaternary amino groups, polyethylene, and reinforcing polyamide mesh (Ltd. “Innovative Enterprise Shchekinoazot,” Tula Region, Russia). The anion exchange resin is polystyrene crosslinked by divinylbenzene. The constant current was applied through the working platinum electrodes at a stepwise rate of 1 × 10^−4^ A/s. The potential drop was measured with two Ag/AgCl electrodes immersed in Luggin capillaries on each side of the membrane under study. The solution flow rate through each compartment was 14 mL/min and was provided from individual tanks.

#### 2.2.4. Distribution of Water over Pore Radii

The structure of membranes was investigated with the method of standard contact porosimetry [23], which is based on capillary equilibrium between the standards with known pore distributions on radii and the samples under investigation. For this purpose, the membrane samples were placed between standards and partially dried. After achieving equilibrium, the samples and standards were weighted. The pore radius corresponding to a given water volume was obtained from porosimetric curves for standards. The porosimetric curves for the investigated samples were presented as dependencies of the water volume distribution in the membrane on the water binding energy or the effective pore radii (*r*).

#### 2.2.5. Fuel Cell Testing

The catalytic ink was obtained via mixing the catalyst (E-TEC-40 on Vulcan XC-42 (40 wt. % Pt)), LF-4SK dispersion (10 wt. % isopropyl alcohol), and water–isopropyl alcohol solvent and sonicating for 1 h. Then, catalytic ink was brushed on the gas diffusion layer (GDL) Toray (280 μm). The electrodes’ loading with Pt was 0.35–0.37 mg Pt/cm^2^. The MEA with a 5 cm^2^ square was formed via hot pressing the GDL and membrane. The cell was tested at 25 °C with air (180 L/h) and H2 (20 L/h) supplied to the cathode and the anode, correspondingly. The relative humidity of the gases was 100%. The MEA was tested according to the next protocol: (i) 2000 cycles of triangular pulses in the potential range of 0.6–1.2 V with the potential scan rate of 100 mV/s; (ii) load current–voltage curves of the MEA (CV-curves) in the step-vise potentiostatic mode in the potential range of 0.05–0.9 V, step value of 50 mV, and duration of every step of 120 s; and (iii) electrochemical impedance spectra (EIS) in the frequency range of 0.5 Hz–500 kHz under 0.5 V loading, and the AC potential amplitude was 30 mV. All electrochemical characteristics of the MEA were studied using the potentiostat–galvanostat Autolab 302N equipped with the FRA32 impedance unit (Metrohm AG, Herisau, Switzerland).

All experiments were performed at 25 °C, and the data were measured at least 3 times and averaged. The experimental error did not exceed 5%.

## 3. Results and Discussion

The main physical–chemical characteristics (thickness l, ion-exchange capacity IEC, water content W_dry_, specific water content nm) of membranes in H+-form are presented in Table 1. As can be seen, in general, the relationship between membrane thickness and its characteristics is absent. The exception is samples with a thickness of 60–65 μm, which have a higher water content. Samples 2–6 have close values of ion-exchange capacity, which is lower than that for Nafion NRE-212.

### 3.1. Conductivity and Diffusion Permeability of the Membranes

**Figure 1:**
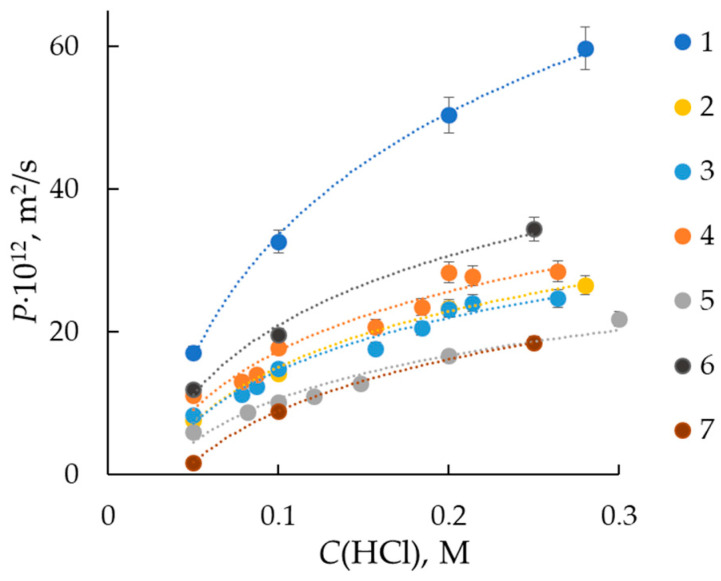
Concentration dependencies of diffusion permeability of Nafion NRE-212 (1) and MF-4SK (2–7) membranes to hydrochloric acid solutions. Curve numbers correspond to sample numbers in Table 1.

The characteristics of MF-4SK perfluorinated membranes with low thickness are measured in comparison with the Nafion NRE-212 (DuPont de Nemour, USA)—the “world leader” in terms of the volume of application in PTEMFC and PEMWE. The concentration dependencies of diffusion permeability coefficients for studied membranes in hydrochloric acid solutions are obtained (Figure 1). The diffusion permeability curves have a typical increasing shape for the ion-exchange membrane in a solution of simple 1,1-electrolyte. If the ion-exchange membrane is presented as a chaotic mixture of gel phase and intergel solution, then the diffusion permeability is determined with the portion and concentration of intergel solution and Donnan sorption of electrolyte with the gel phase [20].

The curves for MF-4SK membranes form one group regardless of the thickness and water content of the membranes, while Nafions membrane have a higher value of diffusion permeability, which is probably caused by their high water content. The gas transport in the membrane could occur in pore space and through crystallite fragments of the membrane. Therefore, the diffusion permeability is proportional to the crossover of liquid and dissolved components, and its value permits the estimation of the gas permeability of the membrane.

**Figure 2:**
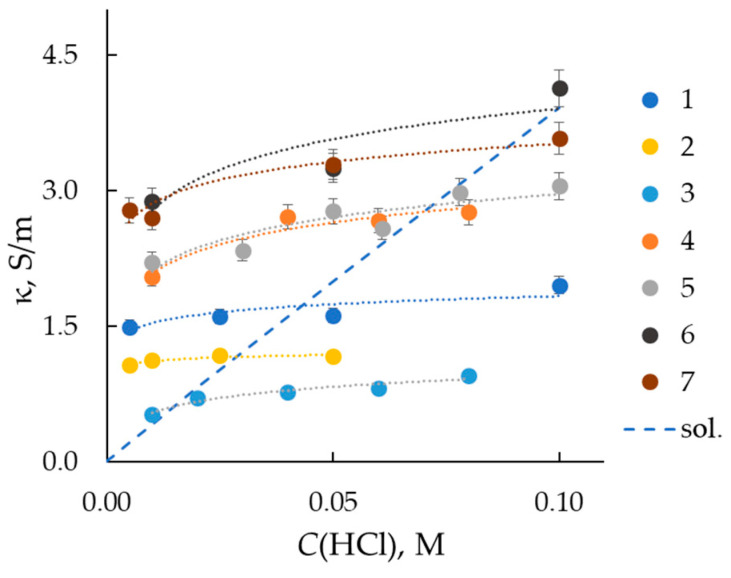
Concentration dependencies of specific conductivity of Nafion NRE-212 (1) and MF-4SK (2–7) membranes in HCl solutions. Curve numbers correspond to sample numbers in Table 1. The dashed line (sol.) is the conductivity of the HCl solution.

Figure 2 shows the concentration dependencies of membrane conductivity in hydrochloric acid solutions. As can be seen, the conductivity of the samples with a greater thickness is higher in comparison with the thinner samples, which is consistent with the results of [18] for Nafion membranes with different thicknesses. As a rule, the resistance of the thin membrane (*R_m_*) is determined as the difference between the resistance of the cell with the membrane (*R_cell_*) and the resistance of the membrane/electrode interface (*R_el/m_*), which corresponds to the resistance of the cell without the membrane [16,24].

(1) $$R_{m}=R_{cell}+R_{el/m},$$

In the case of transversal resistance measurements, Formula (1) corresponds to the serial connection of conductors. If the membrane resistance is measured in contact with an electrolyte solution, the resistance of the solution and the electrode/electrolyte interface can be determined experimentally [25,26]. For mercury-contact cells, this method is inapplicable. To assess the effect of the resistance of the electrode/membrane interface on the total cell resistance, we selected samples from the studied set of membranes that have the same IEC, water content, and differ in thickness (samples Nos. 4 and 5). As can be seen from Figure 2, these samples have similar conductivity values at equal concentrations of the equilibrium hydrochloric acid solution. This indicates an insignificant contribution of the resistance of the mercury/membrane interface to the resistance of the cell.

The curves presented in Figure 2 form two groups: (i) samples Nos. 4–7 with thicknesses from 90 to 300 μm and close values of water content of about 22–23%; (ii) samples Nos. 1–3 with thicknesses about 60–70 μm and higher water content in the range from 30 to 44%. Among membranes with a thickness above 70 µm, the electrical conductivity decreases as their water content decreases. Sample No. 2 of the extrusion membrane has a higher conductivity compared to casting sample No 3. And a close value of diffusion permeability, which makes it most promising for use in fuel cells.

An unusual effect is the lower conductivity of the Nafion NRE-212 membrane in comparison with MF-4SK membrane samples Nos. 4 and 5, while Nafion has higher diffusion permeability and water content.

If the membrane is presented as a chaotic mixture of a swollen polymer (gel) phase and an internal equilibrium solution, then the intersection of the concentration dependences of the membrane and the solution conductivity will allow one to estimate the electrical conductivity of the gel phase of the membrane [20]. When PEMFC and PEMWE operate, the membrane is in conditions of limited humidity and elevated temperature, i.e., in a partially swollen state. Studying the specific electrical conductivity of a membrane in an acid solution permits evaluating the conductivity of the gel phase of the membrane, which includes the hydrated fixed groups and counterions. These fragments of the membrane structure provide the current transfer under restricted humidity. As can be seen from Figure 2, the electrical conductivity of the gel is slightly higher for membranes with a specific moisture content of 13–14 molH_2_O/molSO_3_^−^, which may be due to the facilitated proton hopping between the fixed groups. As expected, the Nafion membrane, which has a higher exchange capacity, has the highest electrical conductivity of the gel phase among thin membranes.

**Figure 3:**
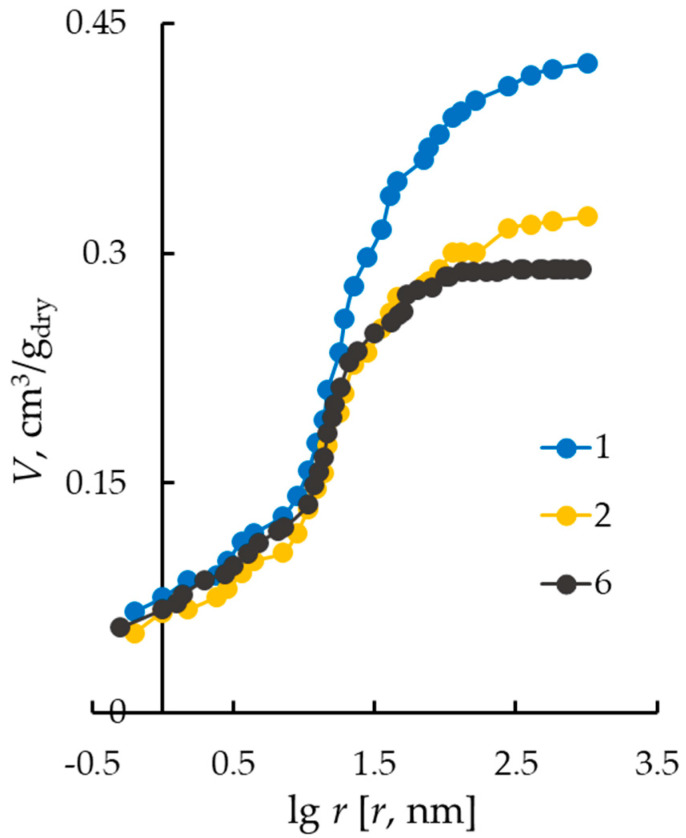
Integral functions of water distribution on the effective pore radii. Curve numbers correspond to sample numbers in Table 1.

The information on electrical transport characteristics is supplemented with an experimental study of the distribution of water over effective pore radii using the standard contact porosimetry method (Figure 3). As can be seen from the figure, the porosimetric curves for membranes with different thickness coincide in the region of micropores (pores with radii less than 10 nm). The main differences in the porometric curve are observed in the region of mesopores (15–100 nm), through which non-selective ion transport is possible. This fact explains the higher diffusion permeability values observed for the Nafion membrane (sample No. 1) compared to MF-4SK (samples Nos. 2 and 6). It could be proposed that the selectivity of the MF-4SK membranes is higher than that of the Nafion membrane.

### 3.2. Voltammetry

**Figure 4:**
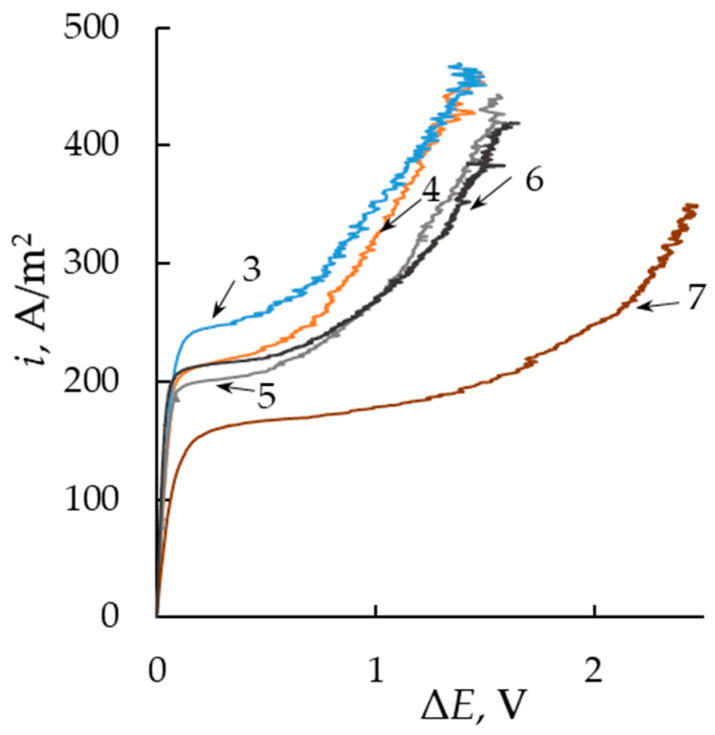
CVCs of MF-4SK membranes in 0.05 mol/L solutions of hydrochloric acid. Curve numbers correspond to sample numbers in Table 1.

To estimate the influence of the membrane thickness on proton transport through the membrane under its polarization with a direct current, we measured the CVCs of membranes in a 0.05 M solution of hydrochloric acid (Figure 4). The presented CVCs have three distinctive regions based on the slopes of the CVC. The ohmic region (first region) is the linear increase in the potential drop with the impressed current. The resistance of the system can be calculated with the inverse of the slope of the tangential line of the ohmic region (*R*_Ohm_). This property is also often called “effective resistance” and includes the membrane resistance and the resistance of the diffusion boundary layers. However, the direct current method does not allow discriminating between individual resistances [27]. The plateau region (second region) can be seen after reaching a limiting current density (*i*_lim_). The value of *i*_lim_ is reached when the depleted layer near the membrane is deficient in ions. The value of *i*_lim_ can be obtained from the experimental data at the intersection of the slopes of the ohmic and plateau regions. The higher this characteristic of the membrane, the higher the efficiency of electromembrane processes with this membrane. According to the Pierse–Gnusin equation [28,29], the *i*_lim_ value depends on the concentration (*C*) and nature of the electrolyte, as well as the differential coefficient (P*) of diffusion permeability and the thickness (*l*) of the membrane.

(2) $$i_{lim}=\frac{DCF}{\left(t_{i}^{*}-t_{i}\right)\delta }+\frac{P^{*}FC}{\left(t_{i}^{*}-t_{i}\right)l},\;P^{*}=P\frac{d\;log\;j}{d\log C},$$

where *D* is the diffusion coefficient of electrolyte in solution, *δ* is the diffusion boundary layer thickness, *t*_i_* and *t*_i_ are the counterion transport numbers in membrane and solution, correspondingly, and *j* is the diffusion flux across the membrane. The thickness of the diffusion boundary layer depends on the hydrodynamic mode and the microstructure of the membrane surface. Since all these membranes have a similar structure and the solution flow rate is the same in all experiments, the thickness of the diffusion layer is equal for all membranes. Investigated membranes possess high selectivity, and *t*_i_* ≈ 1. Therefore, the *i*_lim_ value is directly proportional to the diffusion permeability and inversely proportional to the membrane thickness.

The third region on the CVC is due to the emergence of new mechanisms of ionic mass transport like water splitting and electroconvection. The electrical resistance of the third region (*R*_overlim_) can be determined with the same methodology as that of the ohmic region. There are a variety of factors that impact the shape of a CVC. The length of the plateau region becomes smaller, and the value of *R*_overlim_/*R*_Ohm_ reduces due to the intensification of ion transport to the membrane surface induced by enhanced development of electroconvection or water splitting [27].

**Figure 5:**
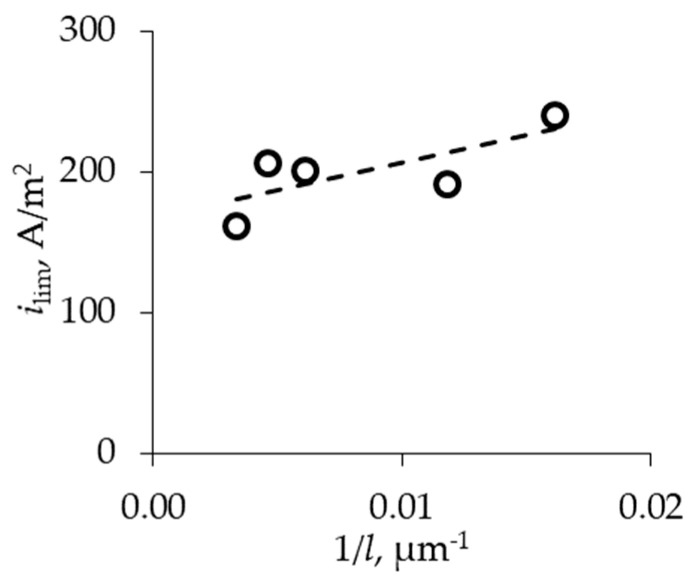
Dependencies of limiting current density on thickness of MF-4SK membranes.

It is found that the limiting current density increases by almost 50% with the reduction in membrane thickness from 300 to 60 μm (Figure 5) due to the higher value of diffusion flux through a thin membrane. In general, the obtained results are in good agreement with the concept that the efficiency of electromembrane processes increases with the decreasing thickness of the membrane.

**Figure 6:**
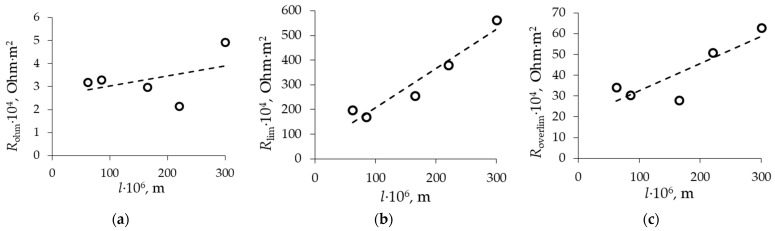
Dependencies of first *R*_Ohm_ (**a**), second *R*_lim_ (**b**), and third *R*_overlim_ (**c**) region slopes of CVCs on membrane thickness.

**Figure 7:**
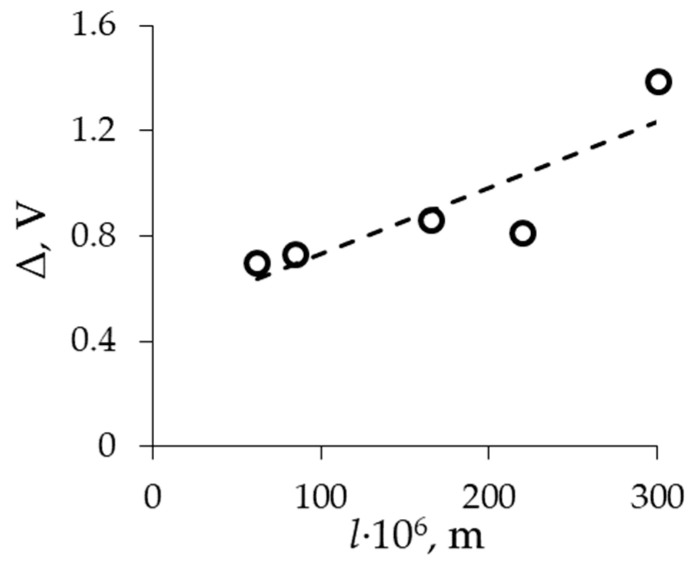
Dependence of limiting current plateau length (Δ) on membrane thickness.

The influence of membrane thickness on the slope of the ohmic region is not observed because the resistance of the diffusion boundary layer mainly contributes to the ohmic resistance of the system (Figure 6). However, the reduction in both the limiting plateau length (Figure 7) and the slope of the limiting region (Figure 6) is observed at currents above the limiting one. In the case of a thinner membrane, the ion flux through the membrane is higher in comparison with the membrane with a higher thickness, which leads to a reduction in the electrical resistance of the membrane as the current density increases.

**Figure 8:**
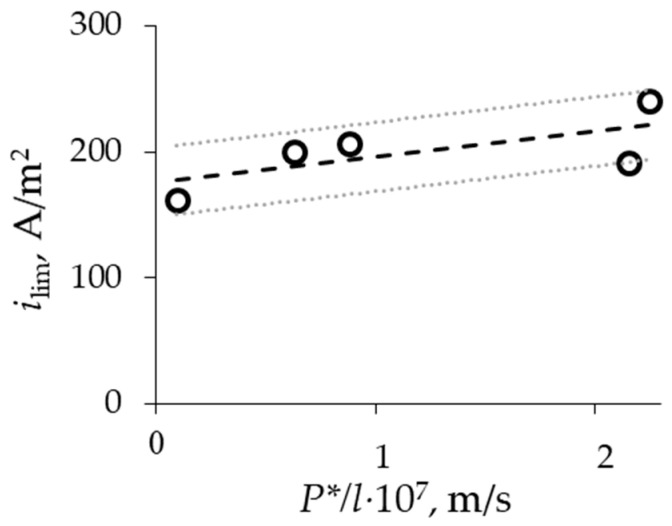
*i*_lim_—P*/*l* for MF-4SK membranes with different thickness.

According to the Pierce–Gnusin equation (Equation (2)), the diffusion flux, which is directed oppositely to the migration one, should influence the limiting current value. Figure 8 demonstrates the dependence of limiting current density on P*/*l*, which is linear according to the Pierce–Gnusin equation.

### 3.3. Behavior of Model Fuel Cell with Different Membranes

**Figure 9:**
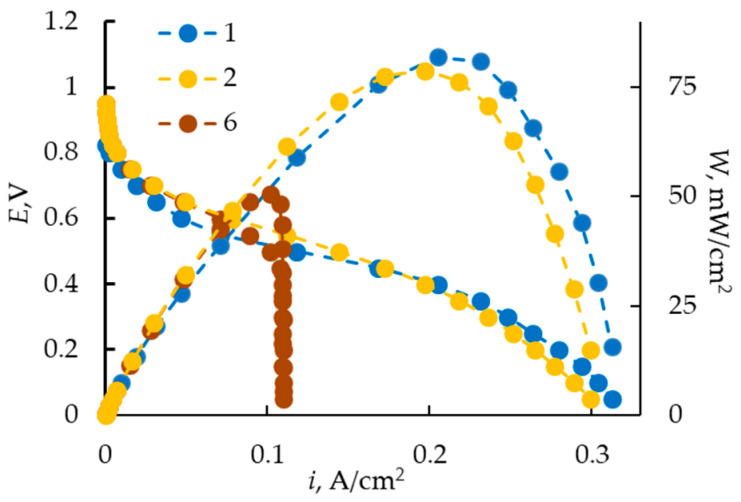
CV and power curves for MEA with MF-4SK membranes. Curve numbers correspond to sample numbers in Table 1.

The electrochemical characteristics of MEA with samples Nos. 1, 2, and 6 are shown in Figure 9. Samples Nos. 2 and 6 are prepared via extrusion and have close values of IEC. So, they differ in thickness and water content. The membrane thickness could influence both MEA resistance and proton transport from anode to cathode. The first effect should manifest itself as an increase in ohmic losses in CV curves, with the second one at the region of diffusion limitations. The slope of the ohmic region is close for all samples, regardless of their thickness and preparation method. The main differences are observed in the diffusion region of the CV curve. 

**Figure 10:**
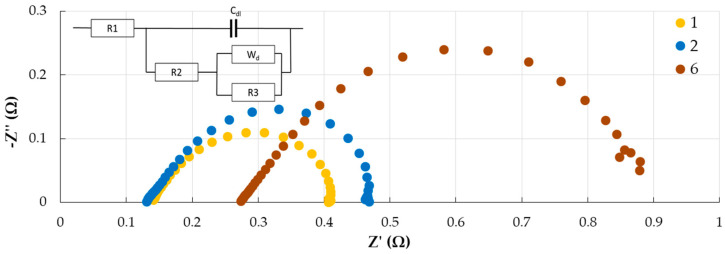
EIS (Nyquist plots) of MEA with MF-4SK membranes under polarization of 0.5 V. Curve numbers correspond to sample numbers in Table 1.

Based on the EIS measured at an external polarization of 0.5 V (Figure 10), the active resistance of the MEA is estimated, which is mainly determined by the resistance of the polymer electrolyte. The calculation of membrane conductivity, assuming that the resistance of the electrodes and membrane/electrode interface is low, shows that its value is 0.9 and 1.2 S/m for the MF-4SK membranes with a thickness of 60 and 220 μm and 1.2 S/m for the Nafion NRE-212 membrane. As a whole, the current range of membrane conductivity does not have a significant effect on the ohmic part of the CV curve of the MEA. The most probable cause of the earlier onset of the limiting state on the CV curve, which is especially pronounced for the extrusion membrane with a thickness of 220 μm, is diffusion limitations associated with proton transport in the membrane.

The EIS are fitted to an equivalent circuit, as presented in Figure 10. The electrochemical impedance spectra contain two arcs: in the high-frequency region, corresponding to the kinetic limitations of the cathode reaction, and in the low-frequency region, corresponding to the diffusion process in the cathode catalytic layer [30]. Diffusion limitations refer to the transport of gases or protons to catalytic centers in the cathode active layer. The equivalent MEA circuit includes the ohmic resistance of the system (R_1_), the kinetic resistance of charge transfer in the cathode reaction (R_2_), and the diffusion resistance to the transport of oxygen and protons to the catalytic centers at the cathode (W_d_+R_3_). Thus, with an increase in the membrane thickness, an increase in diffusion resistance is observed, which corresponds to a low-frequency arch in the EIS. 

It is known that proton could be transported in a cation-exchange membrane via three different mechanisms: the Grotthus mechanism within a hydrogen-bonded water network, the vehicular mechanism, and hopping between neighboring fixed groups, which depends on the thickness of the polymer electrolyte and the overvoltage on the cell [2,31]. To implement the first two mechanisms, the presence in the membrane of a sufficient number of water molecules is required, which are formed at the cathode under the operation of MEA and redistributed throughout the polymer electrolyte volume due to the chemical potential gradient. The third mechanism becomes significant when the membrane dries out. Probably, the increase in diffusion resistance with increasing membrane thickness is due to the drying out of the polymer electrolyte because of the ineffective redistribution of water in its volume. It should be mentioned that despite the lower IEC of Sample No. 2 in comparison with Nafion NRE-212, under the selected experimental conditions, the characteristics of MEA with an extrusion MF-4SK membrane (60 μm) are close to those of Nafion.

## 4. Conclusions

The conductivity, diffusion permeability, and current–voltage curves of perfluorinated membranes of various thicknesses in hydrochloric acid solutions are studied. It is found that diffusion permeability and conductivity are primarily determined with the specific water content of the membranes and increase with their increase rather than with the thickness of the membrane. In the case of the mercury-contact method of measuring the resistance of membranes, the contribution of the electrode–membrane interface resistance can be neglected.

Analysis of the contribution of reverse diffusion through the membrane to the value of the limiting current shows that the Pierce–Gnusin equation is satisfied for perfluorinated membranes, and the contribution of reverse diffusion of the electrolyte to the value of the limiting current density can reach 20%. 

A study of the characteristics of a membrane-electrode assembly with perfluorinated membranes of different thicknesses shows that the thickness of the membrane affects not only the ohmic resistance of the MEA but also the diffusion limitations of proton transport, which is manifested in the earlier onset of the limiting state on the current–voltage curves of the MEA with a thick membrane compared to with thin membranes.

## Figures and Tables

**Table 1:** Physical–chemical characteristics of membranes.

No	Membrane	l, μm	IEC, mmol/g_dry_	W_dry_, %	n_m_, mol H_2_O/mol SO_3_^−^
1	Nafion NRE-212	65 ± 2	1.05 ± 0.05	44 ± 0.5	23 ± 0.5
2	MF-4SK (extrusion)	60 ± 2	0.93 ± 0.05	34 ± 0.5	20 ± 0.5
3	MF-4SK (cast)	60 ± 2	0.98 ± 0.05	31 ± 0.5	18 ± 0.5
4	MF-4SK (extrusion)	90 ± 2	0.93 ± 0.05	22 ± 0.5	13 ± 0.5
5	MF-4SK (extrusion)	170 ± 2	0.93 ± 0.05	22 ± 0.5	13 ± 0.5
6	MF-4SK (extrusion)	240 ± 2	0.91 ± 0.05	23 ± 0.5	14 ± 0.5
7	MF-4SK (extrusion)	300 ± 2	0.65 ± 0.05	23 ± 0.5	20 ± 0.5

## Data Availability

The data presented in this study are available on request from the corresponding author.
